# Ferroptosis-associated lncRNA prognostic signature predicts prognosis and immune response in clear cell renal cell carcinoma

**DOI:** 10.1038/s41598-023-29305-5

**Published:** 2023-02-06

**Authors:** Jiayi Lai, Shiqi Miao, Longke Ran

**Affiliations:** 1 Department of Bioinformatics, The Basic Medical School of Chongqing Medical University, Chongqing, 400016 China; 2 Department of Biochemistry and Molecular Biology, The Basic Medical School of Chongqing Medical University, Chongqing, China; 3 Department of Biochemistry and Molecular Biology, The Basic Medical School of Chongqing Medical University, Chongqing, China

**Keywords:** Biochemistry, Biological techniques, Cancer, Molecular biology

## Abstract

Clear cell Renal Cell Carcinoma (ccRCC), the most deadly and life-threatening tumor in the urinary system, has a dismal prognosis and a high risk of metastasizing. Regulation of ferroptosis is a prospective therapeutic target to eradicate malignant cells. Our objective was to seek ferroptosis-associated long non-coding RNAs (FALs) and developed a prediction signature for ccRCC. We extracted transcriptome data and clinical information from The Cancer Genome Atlas (TCGA) databases. Ferroptosis-associated genes (FAGs) were obtained from FerrDb database. A ferroptosis-associated lncRNA prognostic signature (FLPS) of ccRCC was generated utilizing univariate Cox regression, least absolute shrinkage and selection operator (LASSO), and multivariate Cox regression, sequentially, based on 8 lncRNAs (LINC00460, AC124854.1, AC084876.1, IGFL2-AS1, LINC00551, AC083967.1, AC073487.1, and LINC02446). The signature's independent predictive value for ccRCC was demonstrated using univariate and multivariate regression analysis (P < 0.05). Subsequently, by combining independent predictive factors, a prognostic nomogram was established. Immunity analysis proclaimed a striking difference in terms of cells, function, checkpoints, and ESTIMATE scores between low- and high-risk groups. Overall, the innovative signature of ferroptosis-associated signatures may have a considerable effect on the immune response and prognosis for ccRCC.

## Introduction

In 2022, The United States is projected to arise 79,000 cases and 13,920 mortality of kidney cancer^1^. Renal Cell Carcinoma (RCC) represents one of the most devastating carcinomas in the urinary system^2^. In addition, papillary RCC (pRCC) and chromophobe RCC (chRCC) are further RCC subtypes^3^. Among these three subtypes, the most common RCC type, Clear cell Renal Cell Carcinoma (ccRCC), comprises 75% of all kidney cancer diagnoses and is also the most invasive subtype with a high metastasis risk and recurrence rates^4,5^. Although ccRCC can be eradicated by local or full nephrectomy, approximately 40% of patients with advanced tumors eventually progress to metastasis as a consequence of surgical intervention^6^. Therefore, it is imperative to do additional research on the carcinogenesis of ccRCC and identify emerging biomarkers and promising therapeutic targets that will contribute to the effective treatment of ccRCC patients.

In recent decades, a rapid increase in research on ferroptosis has arisen. Ferroptosis was first introduced by Dixon^7^ in 2012, which is an original kind of regulatory cell death (RCD) with iron-dependent, defined by the intracellular buildup of reactive oxygen species (ROS)^6^. Compared to apoptosis, necrosis, autophagy, and other types of cellular death, ferroptosis is unique^8^. It is primarily characterized by morphological alterations, thus, the increased mitochondrial membrane density, the disappeared mitochondrial crest, and the ruptured mitochondrial outer membrane during ferroptosis^7,9,10^. The imbalance of lipid reactive oxygen generation and degradation in cells are the primary inducers of ferroptosis^11^. When the antioxidant capacity of cells is declined, the accumulation of ROS can result in oxidative cell death, that is, ferroptosis. Ferroptosis could be induced by several compounds. It occurs in various signaling pathways, however, upstream pathways exert a direct or indirect impact on the glutathione peroxidase (GPXs) activity, weaken cell antioxidant capacity, leading to increased lipid peroxidation, lipid increased ROS, and causing ferroptosis^11^. Ferroptosis has attracted prominence as a research topic recently since it can trigger tumor cells to perish and its suppression can prevent the progression of neurodegenerative diseases. Regardless of the strong correlation between ferroptosis and tumor cells, it is yet unexplained how precisely ferroptosis contributes to the formation and treatment of cancer.

Functional genomics investigations have uncovered enormous quantities of lncRNAs. LncRNAs, which have a length of more than 200 nucleotides, are a subclass of non-coding RNA^12^. Importantly, it has been demonstrated that lncRNAs frequently act as master regulators of gene expression. As a consequence, a wide range of biological processes, as well as pathological processes, such as cancer, depend heavily on lncRNAs^12,13^.

Hence, we deployed bioinformatic analysis to mine probable dysregulated lncRNAs involved in ferroptosis. We acquired KIRC datasets through The Cancer Genome Atlas (TCGA) for this investigation. Eight ferroptosis-associated lncRNAs of prognostic significance were discovered and a prognostic lncRNA signature was developed which could be applied to identify prospective treatment targets. Immune infiltration analysis revealed the expression of immune checkpoint molecules varied widely across these two risk groups, which indicated that immune checkpoint inhibitors may be advantageous to high-risk ccRCC. Here, a potential FA-DEL-based predictive model for patients with ccRCC was developed, which might be applied to estimate prognosis and select patients for immunotherapy.

## Results

### Participants

**Figure 1:**
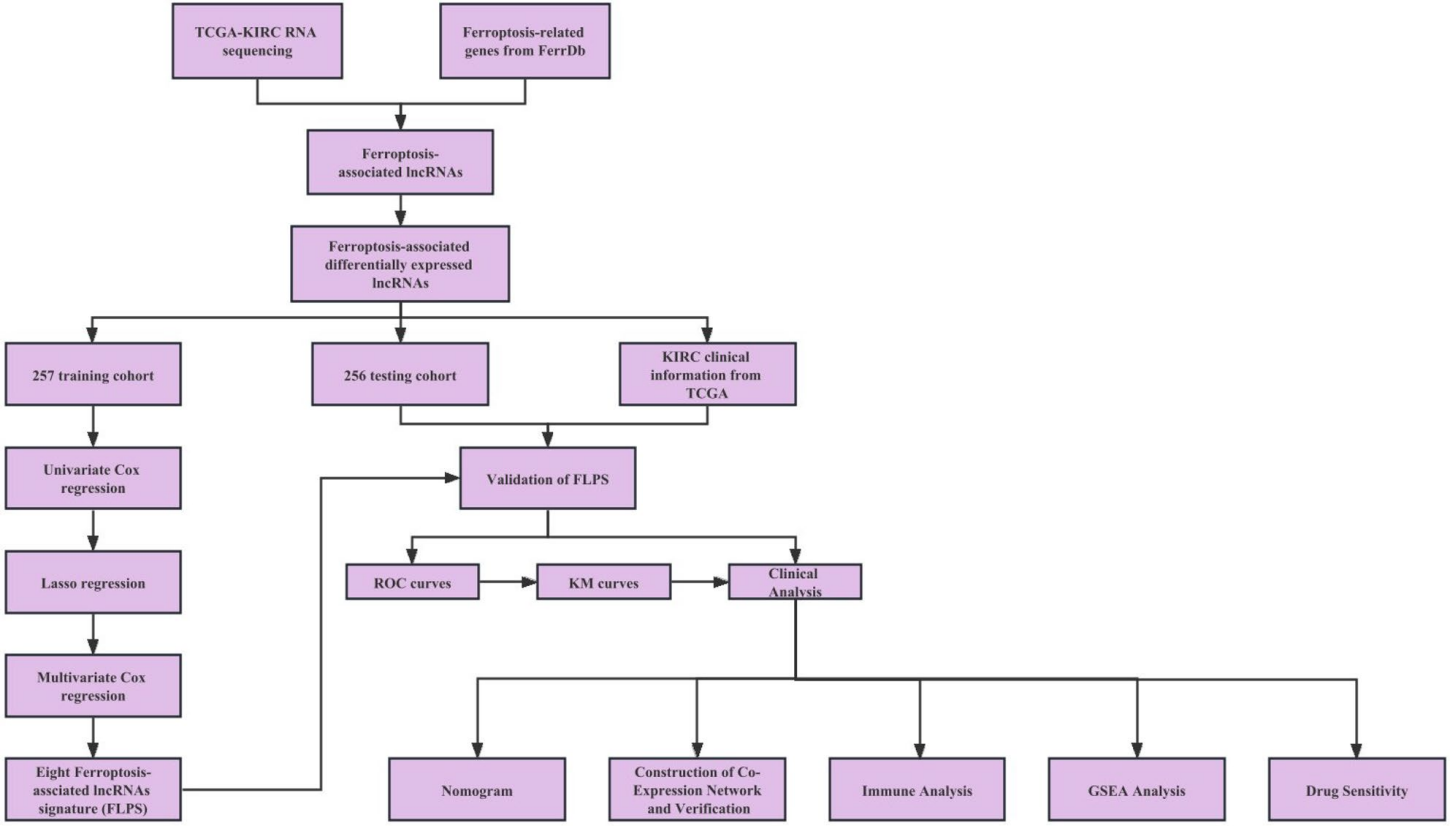
Flowchart of the study.

Our study’s flowchart was visible in Fig. 1. For this study, 513 patients with ccRCC were recruited, including 257 cases from the experimental cohort and 256 cases from the validation cohort. The comprehensive clinicopathological information of the experimental cohort and training cohort from TCGA-KIRC datasets are exhibited in Table 1.

**Table1:** Clinicopathological characteristics of ccRCC patients.

	MALE	164 (32%)	173 (33.7%)
Grade, n (%)	G1	9 (1.8%)	3 (0.6%)
	G2	108 (21.1%)	111 (21.6%)
	G3	99 (19.3%)	102 (19.9%)
	G4	39 (7.6%)	34 (6.6%)
	Unknown	2 (0.4%)	6 (1.2%)
Tumor stage, n (%)	Stage I	125 (24.4%)	130 (25.3%)
	Stage II	23 (4.5%)	33 (6.4%)
	Stage III	65 (12.7%)	52 (10.1%)
	Stage IV	44 (8.6%)	38 (7.4%)
	Unknown	0 (0%)	3 (0.6%)
T, n (%)	T1	130 (25.3%)	131 (25.5%)
	T2	31 (6%)	37 (7.2%)
	T3	90 (17.5%)	83 (16.2%)
	T4	6 (1.2%)	5 (1%)
M, n (%)	M0	205 (40%)	202 (39.4%)
	M1	40 (7.8%)	38 (7.4%)
	Unknown	12 (2.3%)	16 (3.1%)
N, n (%)	N0	116 (22.6%)	113 (22%)
	N1	7 (1.4%)	9 (1.8%)
	Unknown	134 (26.1%)	134 (26.1%)

### Investigation of ferroptosis-associated lncRNAs with differentially expressed expression

**Figure 2:**
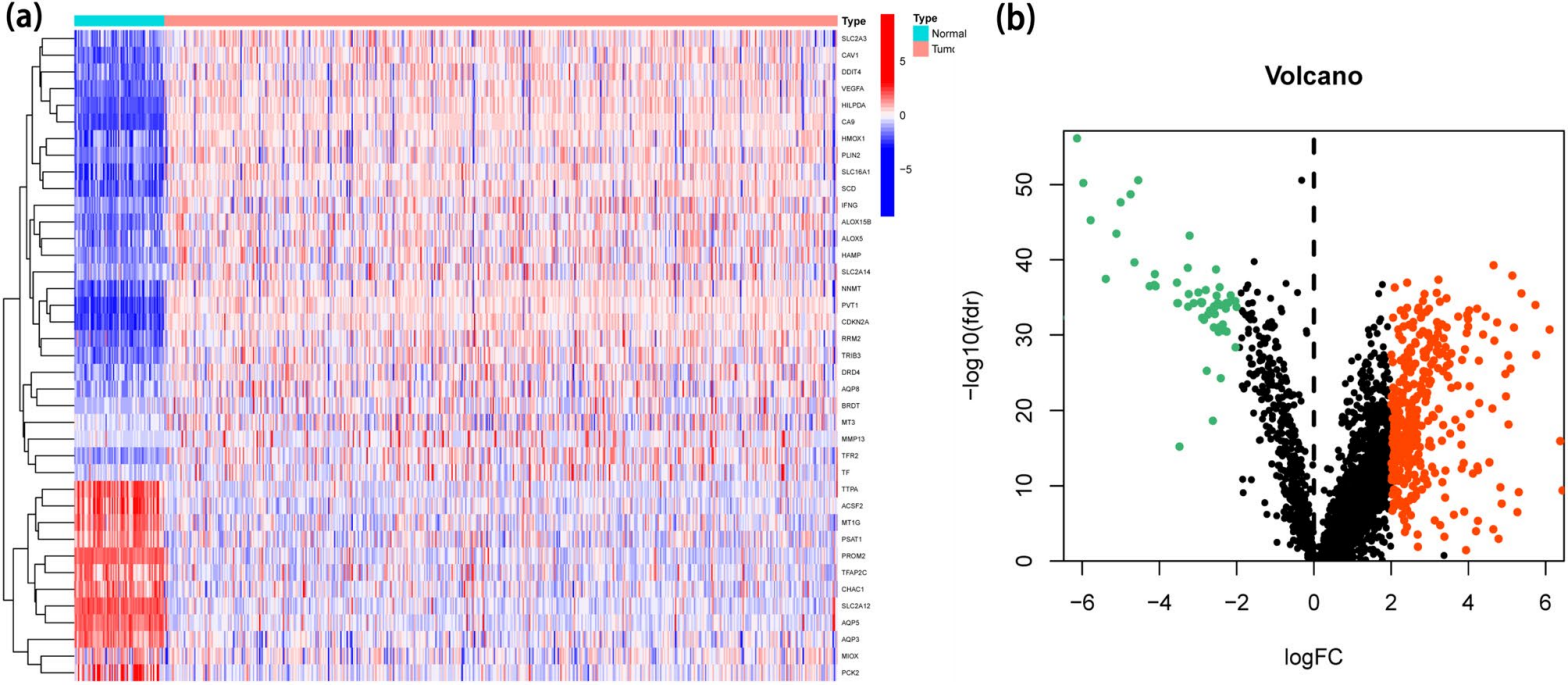
Identification of FA-DEGs and FA-DELs in ccRCC patients. (**a**) Heatmap for 39 FA-DELs. (**b**) The volcano plot of 433 FA-DELs.

We retrieved the TCGA-KIRC dataset and downloaded FAGs from FerrDb. Employing Pearson's correlation analysis, the relationship between 384 FAGs and FALs was assessed. We screened for 2268 FA-DELs. Additionally, the differentially expressed analysis in 384 FAGs and 2268 FA-DELs was carried out in accordance with the established cutoff value of |log2FC| > 2, FDR < 0.05. Then, we acquired 39 FA-DEGs and 433 FA-DELs in total (Fig. 2a, b).

### Development and verification of prognosis FLPS

**Figure 3:**
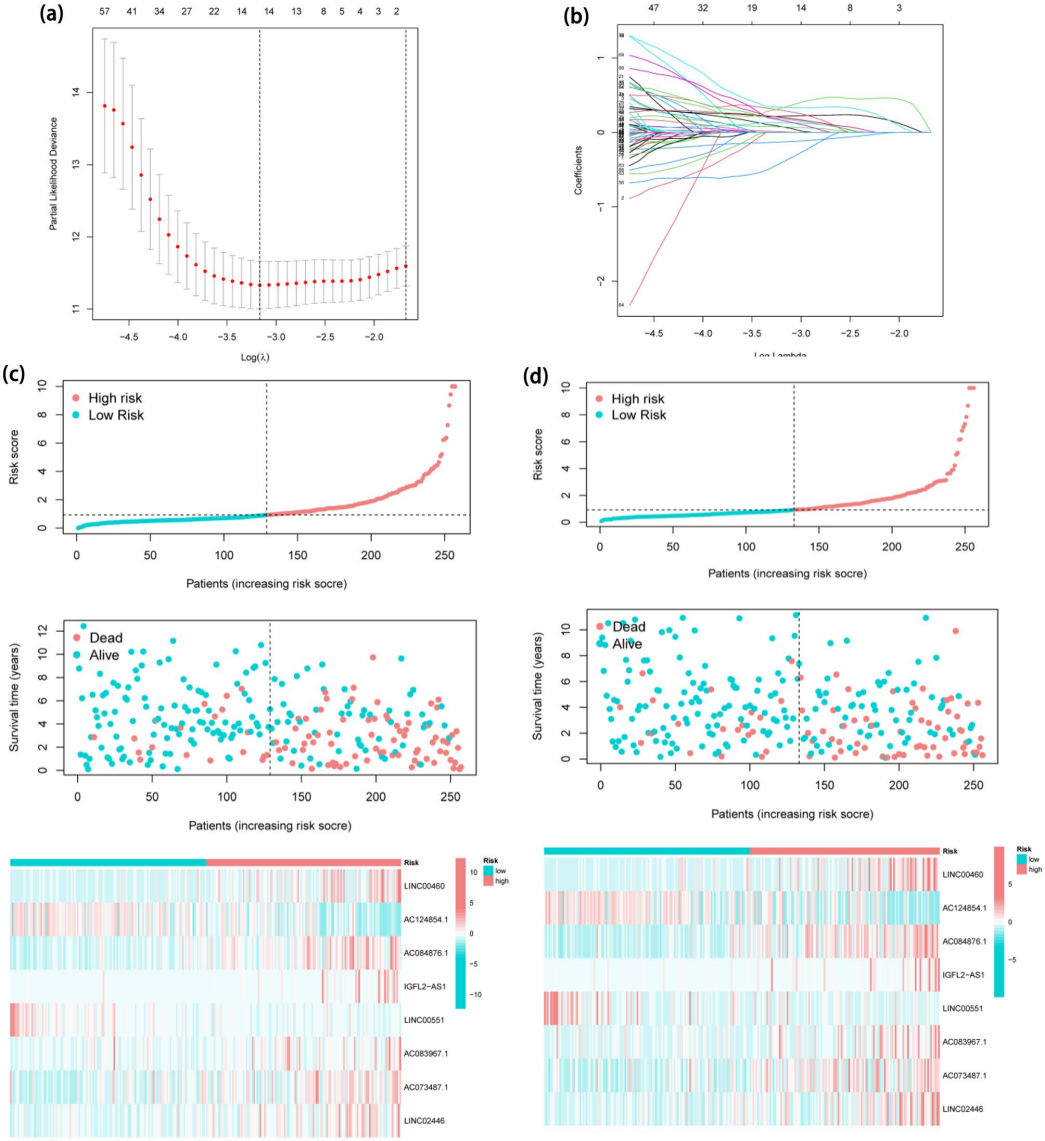

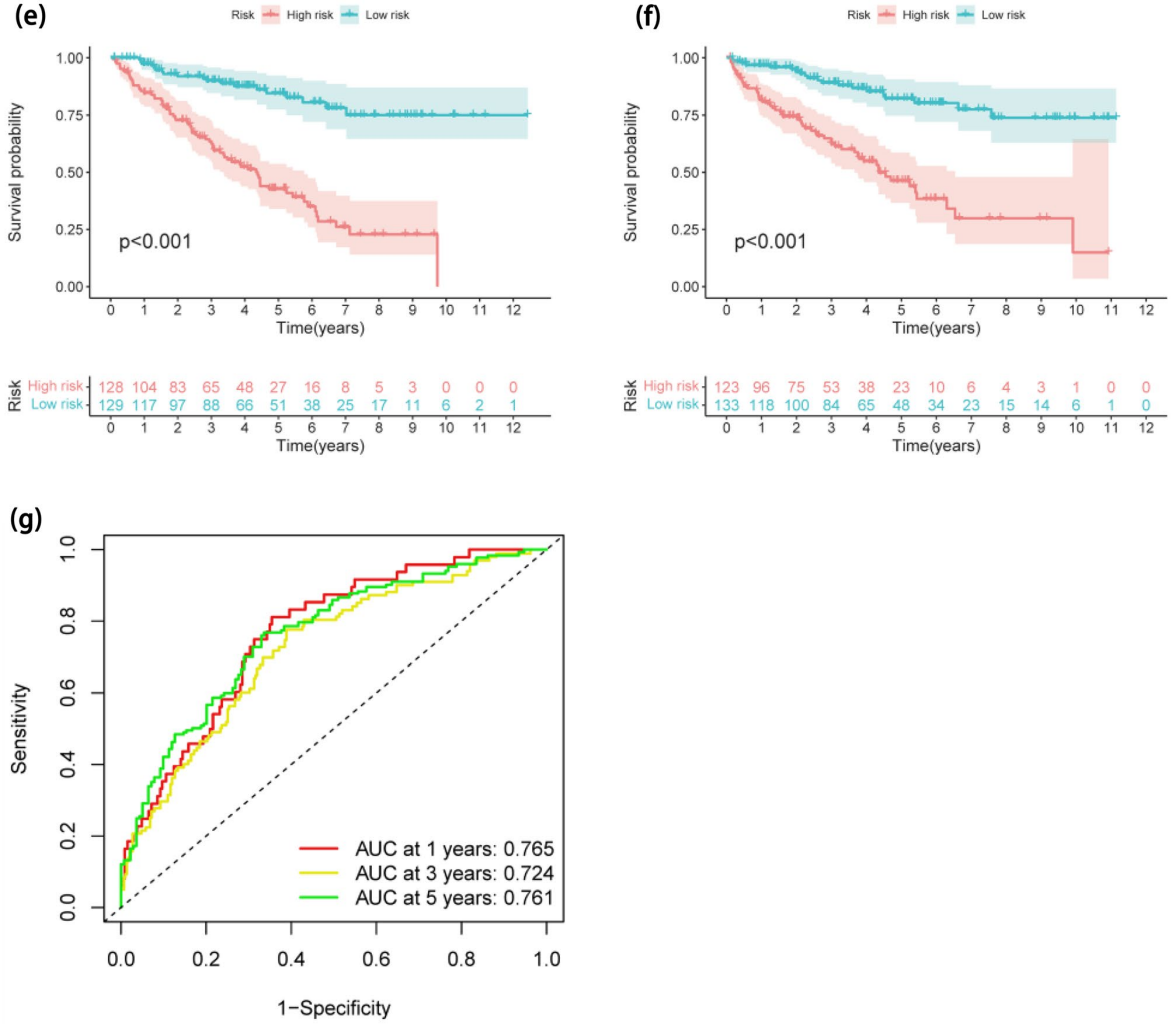
Establishment and verification of FLPS. (**a**) The vertical black line in the figure denotes the ideal log λ value. (**b**) In the LASSO coefficient profile of FALs, each line denotes a distinct lncRNA. (**c**) The risk score, survival status, and heatmap in experimental set. (**d**) The risk score, survival status, and heatmap in validation set. (**e**) KM curves for survival probability in experimental cohort (P < 0.001). (**f**) KM curves for survival probability in validation cohort (P < 0.001). (**g**) The AUC values for prediction of survival rates of ccRCC at 1, 3, and 5 years.

433 FA-DELs were uncovered and 97 significant FA-DELs were revealed by univariate regression analysis (Supplementary Table S2), which were incorporated in Lasso regression analysis, subsequently. After screening, 14 FA-DELs remained (Fig. 3a, b). Following that, we conducted multivariate Cox regression. Overall, 8 FA-DELs (LINC00460, AC124854.1, AC084876.1, IGFL2-AS1, LINC00551, AC083967.1, AC073487.1, LINC02446) were discovered to be independent predictive factors of ccRCC, were displayed in Table 2. Hence, we determined risk scores and developed an FLPS.

**Table 2:** Multivariate Cox regression analysis of ferroptosis-associated lncRNA.

Ferroptosis-associated lncRNA	coef
LINC00460	0.244157135
AC124854.1	− 0.169947947
AC084876.1	0.608238496
IGFL2-AS1	0.251676786
LINC00551	− 1.929193729
AC083967.1	0.446834468
AC073487.1	0.599290559
LINC02446	0.305102052

The following was the risk score equation:

$$\begin{aligned} & \left( {0.{2441} \times {\text{LINC}}00{46}0} \right)\, + \left( { - 0.{1699} \times {\text{AC124854}}.{1}} \right)\, + \,\left( {0.{6}0{82} \times {\text{AC}}0{84876}.{1}} \right) \, \\&\quad + \left( {0.{2516} \times {\text{IGFL2}} - {\text{AS1}}} \right)\, + \, \left( { - {1}.{9291} \times {\text{LINC}}00{551}} \right) + \left( {0.{4468} \times {\text{AC}}0{83967}.{1}} \right)\, \hfill \\&\quad + \left( {0.{5992} \times {\text{AC}}0{73487}.{1}} \right)\, + \, \left( {0.{3}0{51} \times {\text{LINC}}0{2446}} \right). \hfill \\ \end{aligned}$$

Figure 3c displays the risk score curve, the survival status, and the heatmap of expression profiles of the 8 FA-DELs. The aforementioned analyses were also used to further validate the FLPS's performance utilizing verification cohort at the same time. Just as we anticipated, in the verification cohort, a semblable trend was found (Fig. 3d). KM analysis showed that the high-risk FLPS was connected to inferior survival than low-risk FLPS (P < 0.001), in experimental and validation sets (Fig. 3e, f). The novel FLPS’s AUC predictive value for 1, 3, and 5-year survival rates were 0.765, 0.724, and 0.761, sequentially (Fig. 3g).

### Assessment of the FLPS as an independent a predictive factor for ccRCC and development of predictive nomogram

**Figure 4:**
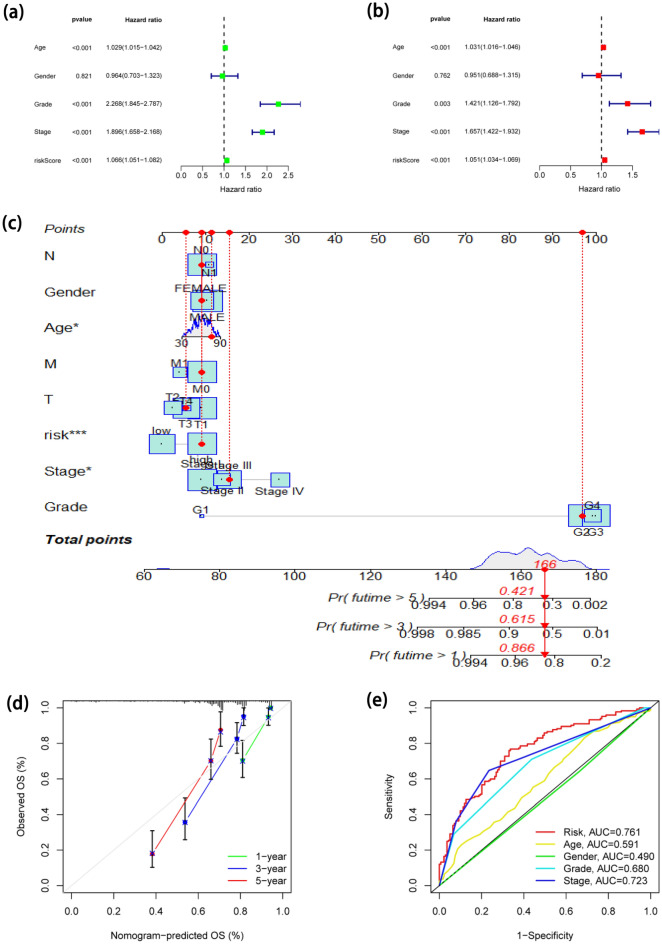
Evaluation of FLPS and development of FLPS-based nomogram. (**a**) Univariate Cox regression analysis for the OS of ccRCC. (**b**) Multivariate Cox regression analysis for the OS of ccRCC. (**c**) A nomogram based on all independent predictive variables. (**d**) Calibration curves for the nomogram. (**e**) ROC curves for predicting OS at 5 years.

Fortunately, the outcomes revealed that FLPS was an independent predictive variable, shown in Fig. 4a, b. The innovative FLPS and clinicopathologic traits were combined to develop a hybrid nomogram that was accurate and stable, allowing it to be used for the clinical care of ccRCC (Fig. 4c). Additionally, calibration curves demonstrated that our proposed nomogram for forecasting the survival of ccRCC had a favorable adaptation (Fig. 4d). ROC curve analysis was developed for the purpose of forecasting overall survival (OS) at 5 years by risk scores, age, gender, grade, and stage (Fig. 4e).

### The correlation of FLPS and clinicopathologic characteristics

**Figure 5:**
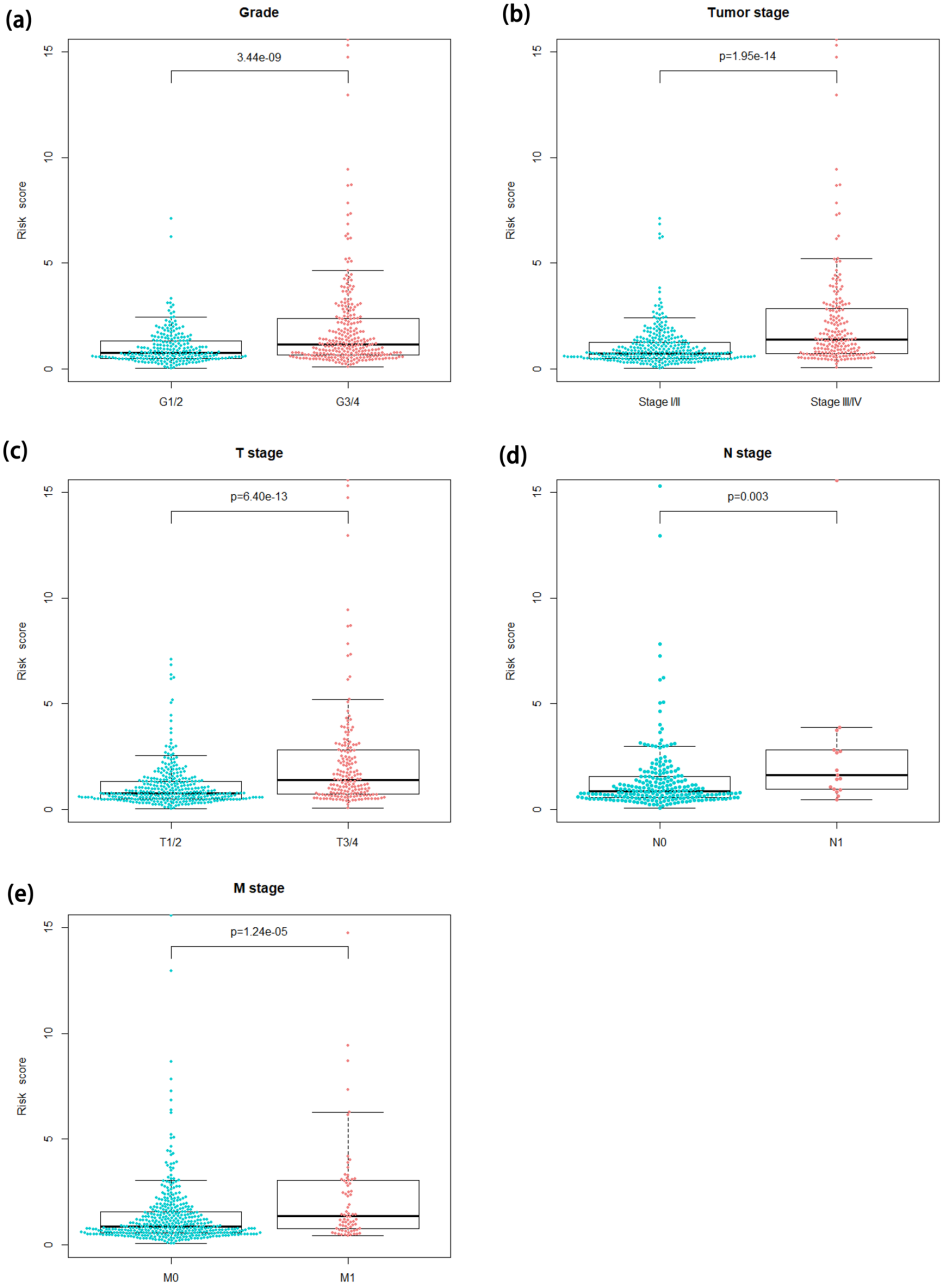
The correlation analysis of FLPS and clinicopathologic characteristics. (**a**) Grade (P = 3.44e−09). (**b**) Tumor stage (P = 1.95e−14). (**c**) T stage (P = 6.40e−13). (**d**) N stage (P = 0.003). (**e**) M stage (P = 1.24e−05).

According to the Wilcoxon rank-sum test, the risk score raised with the development of grade (P = 3.44e−09), tumor stage (P = 1.95e−14), T stage (P = 6.40e−13), N stage (P = 0.003), and M stage (P = 1.24e−05), which suggested that lncRNAs enrolled in FLPS can impact the progression, malignancy, as well as survival outcome of ccRCC (Fig. 5a–e).

### Establishment of a co-expression network and critical LncRNAs verification

**Figure 6:**
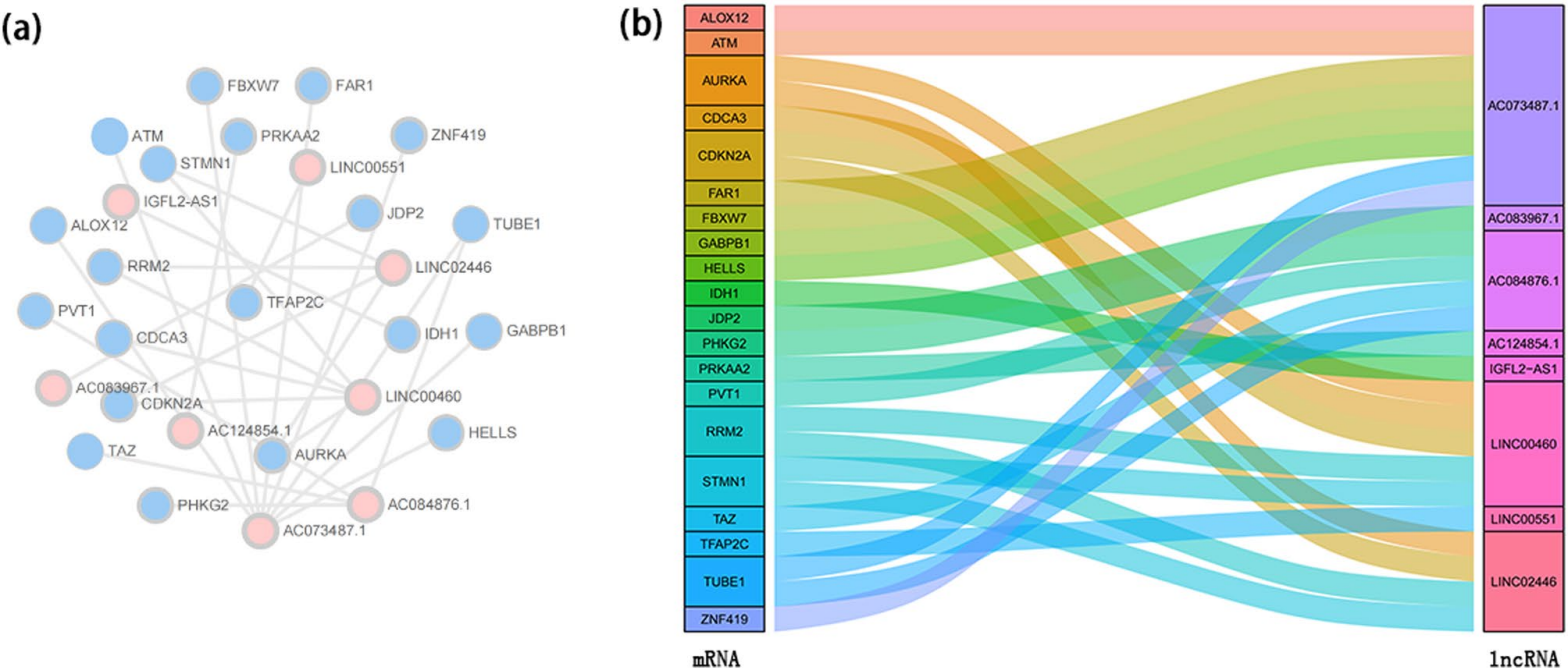
Construction of a co-expression network. (**a**) The co-expression network of 8 FALs and 20 FAGs. (**b**) The Sankey diagram manifested how 8 FALs and 20 FAGs were connected.

The co-expression network of 8 FALs and 20 FAGs is exhibited in Fig. 6a (Supplementary Table S3). The Sankey diagram was conducted to manifest the internal relation between 8 FALs and 20 FAGs, which is shown in Fig. 6b. According to the results of differentially expressed analysis and relevant literature^14–17^, LINC00460 and LINC00551 were selected for further validation. LINC00460 expression was substantially higher in ccRCC (logFC = 5.039969, P = 1.74e−19), however, LINC00551 was downregulated in ccRCC (logFC = − 3.51939, P = 1.62e−36).

**Figure 7:**
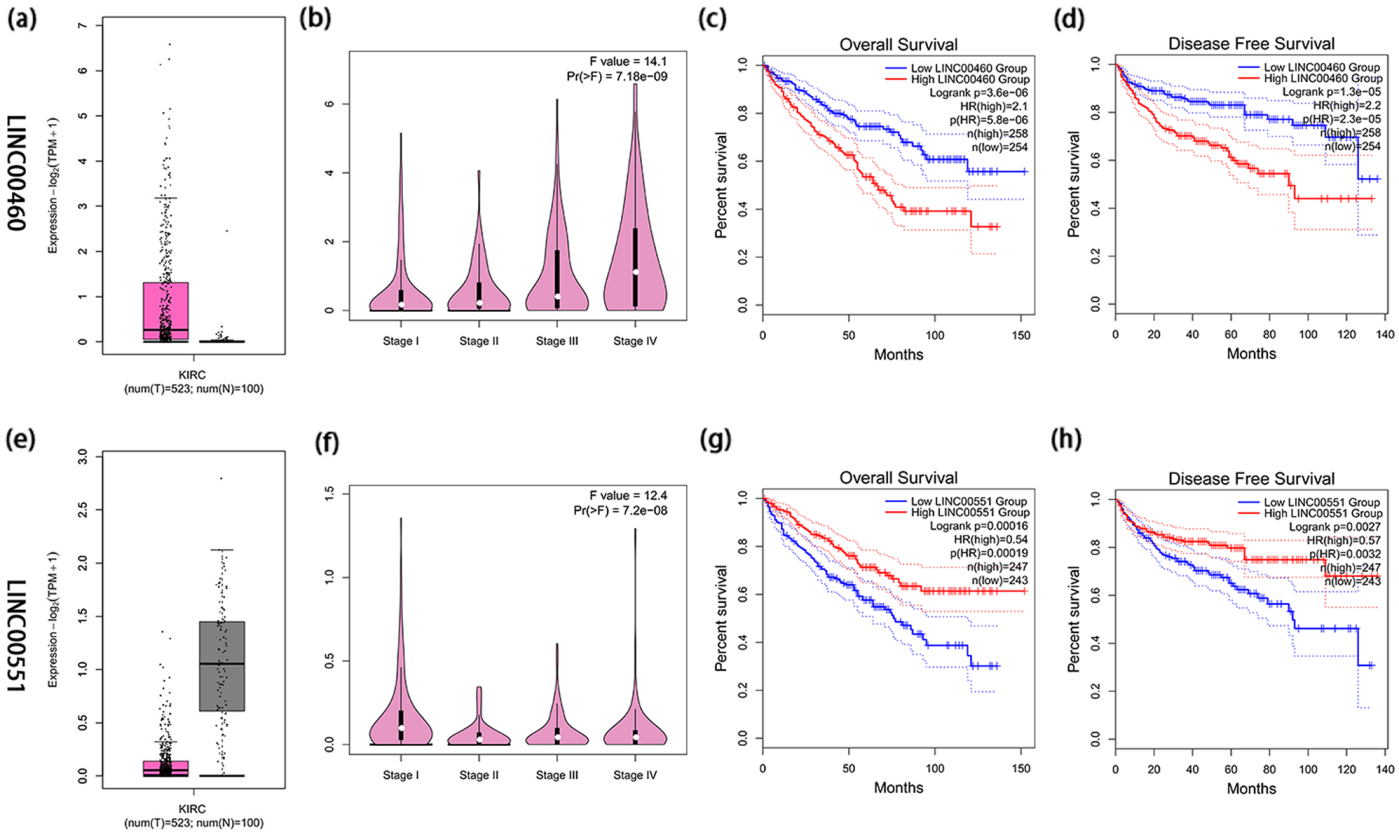
Confirmation of expression and prognosis of 2 FALs (LINC00460 and LINC00551) from the GEPIA2 database. (**a**, **e**) The expression of LINC00460 and LINC00561 in tumor and ccRCC tissues, respectively, the purple color represents ccRCC tissues while the grey color represents normal tissues. (**b**, **f**) LINC00460 expression enhanced with the development of stages (Pr = 7.18e−09), while, with the progression of stages, LINC00551 expression decreased (Pr = 7.2e−08). (**c**, **d**, **g**, **h**) The high expression level of LINC00460 and low expression of LINC00551 were all linked to a poor prognosis (all P < 0.05).

GEPIA2 was performed to examine the expression as well as survival outcomes of two FALs. Figure 7a and e, respectively, depict the expression of LINC00460 and LINC00551 in ccRCC and normal tissues. As it is exhibited in Fig. 7b and f, LINC00460 increased with the improvement of tumor stage (Pr = 7.18e−09), while LINC00551 expression decreased with the increase of tumor stage (Pr = 7.2e−08), which indicated that two FAGs were closely linked to the development of the tumor. A worse prognosis for patients with ccRCC was related to high LINC00460 expression (P < 0.05) (Fig. 7c, d), contrarily, high LINC00551 expression was concerned with a positive outcome of ccRCC (P < 0.05) (Fig. 7g, h).

**Figure 8:**
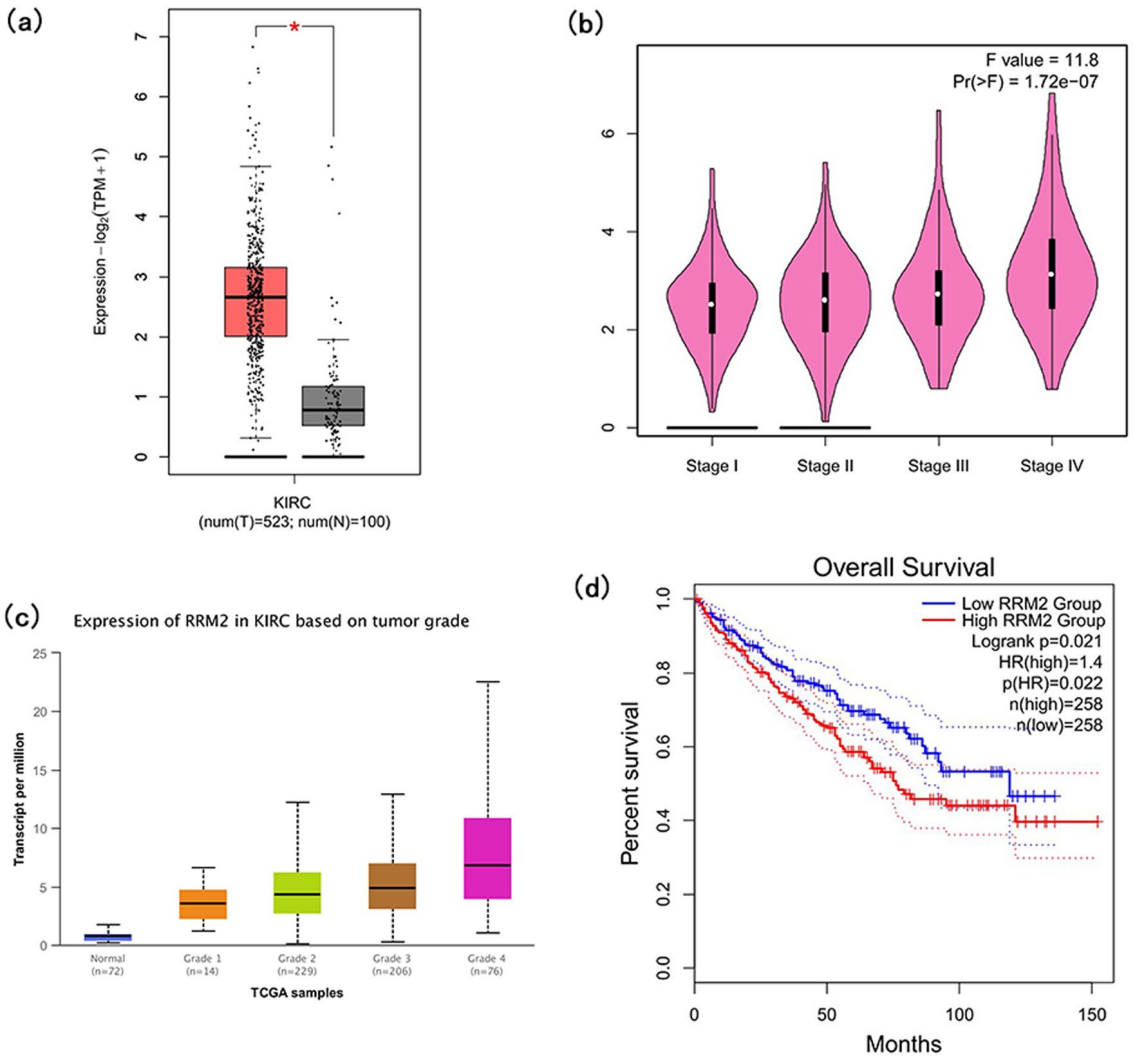
Confirmation of expression and prognosis of RRM2 from GEPIA2 and UALCAN databases. (**a**) RRM2 expression in tumor and ccRCC tissues (P < 0.05), the red indicated ccRCC tissues and the grey indicated normal tissues. (**b**) The expression of RRM2 increased with the advancement of the tumor stage (Pr = 1.72e−07). (**c**) RRM2 expression in KIRC according to tumor grade. (**d**) The high RRM2 was connected to the poor prognosis of individuals with ccRCC (P < 0.05).

By means of correlation analysis and corresponding literature^18,19^, the target gene RRM2 was elected for further verification by GEPIA2 and UALCAN databases. RRM2 was shown to be elevated in ccRCC (P < 0.05) when the expression of RRM2 in ccRCC and normal tissues is displayed in Fig. 8a. RRM2 expression was enhanced with the advancement of tumor stage and tumor grade (Fig. 8b, c), suggesting that RRM2 was associated with the progression of ccRCC. Furthermore, according to Fig. 8d, individuals with ccRCC who had high RRM2 expression had a negative prognosis (Logrank p = 0.021).

### GSEA analysis

**Figure 9:**
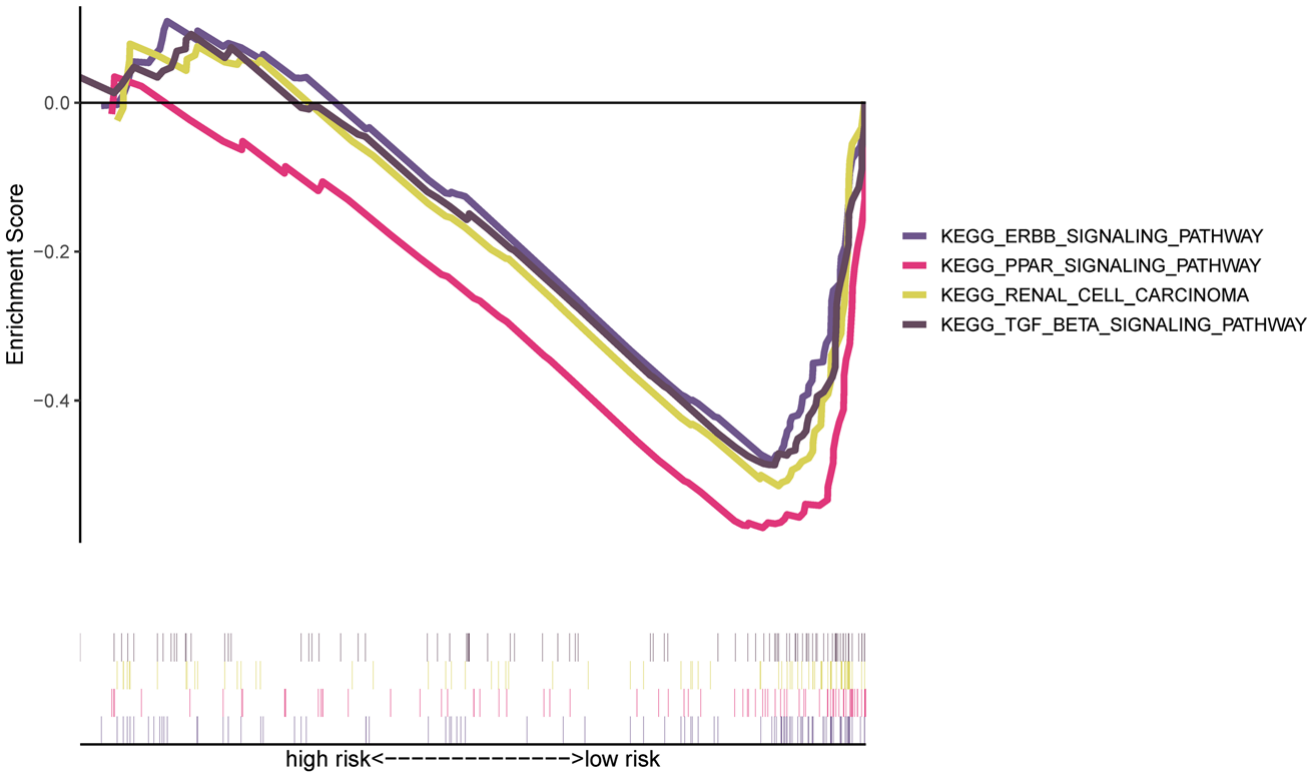
GSEA of FLPS.

The low-risk group, which was enriched in several relatively major tumor signaling pathways (Supplementary Table S4), showed substantial enrichment in a variety of related pathways according to GSEA analysis. For instance, the TGF BETA, PPAR, as well as ERBB signaling pathways (Fig. 9). The biological importance of FLPS in the formation of ccRCC was therefore verified by GSEA enrichment.

### The FLPS correlates with immune infiltration landscape

**Figure 10:**
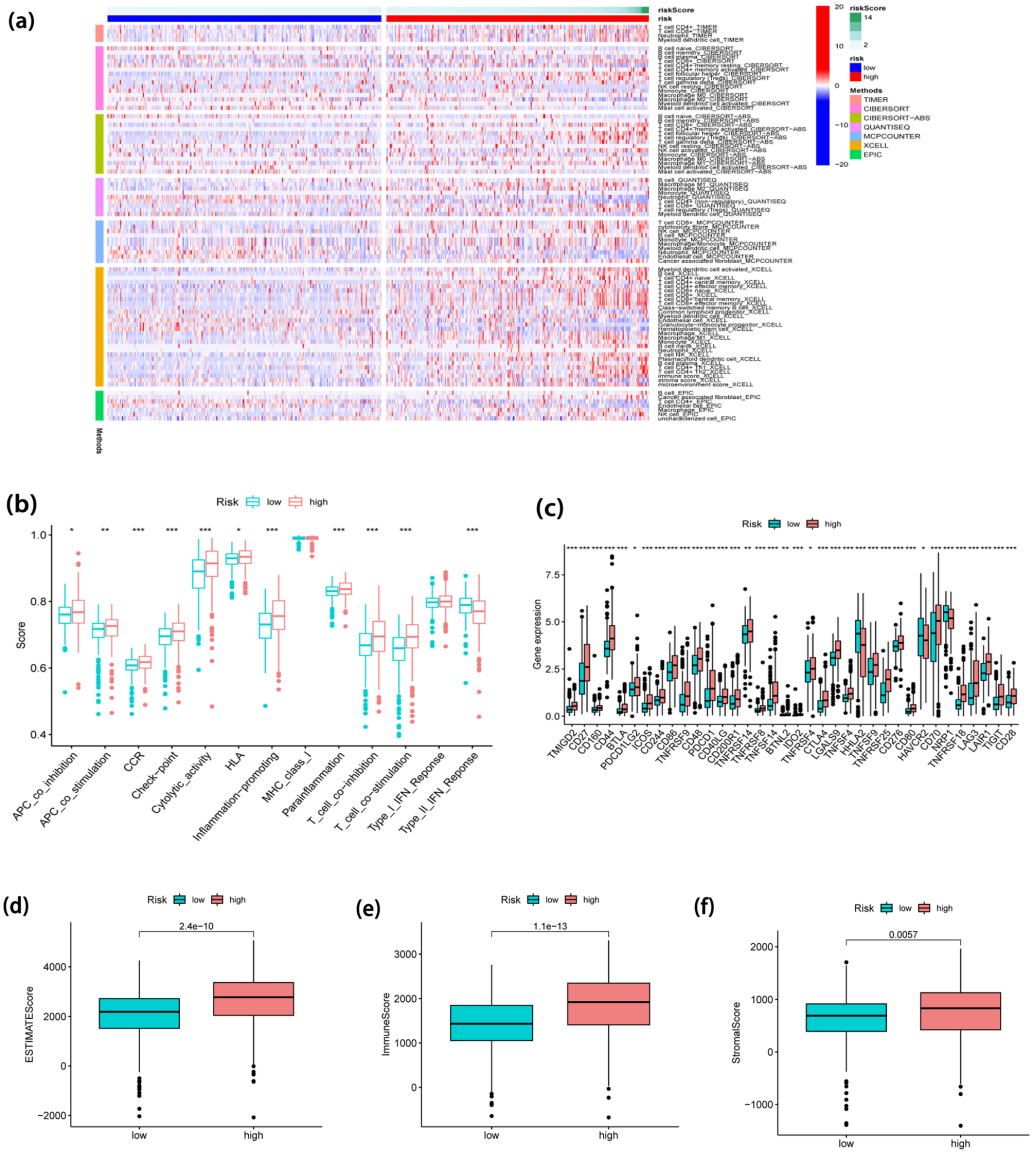
The relationship between FLPS and immunological infiltration landscape. (**a**) Heatmap for tumor-infiltrating immune cell types. (**b**) The immune-associated activity. (**c**) Expression of the immune checkpoint. (**d**–**f**) Comparison of ESTIMATE, immune, and stromal scores in two groups.

The heatmap of distinct immunological cells abundance (immune response) based on 6 different algorithms is shown in Fig. 10a. A number of immunological cells were relatively related to high-risk group, including T cell CD4+ at TIMER, T cell regulatory (Tregs) at QUANTISEQ, cytotoxicity score at MCPcounter, T cell NK at XCELL, and Macrophage at EPIC. The immune function ssGSEA scores were implemented to examine the connection between risk score and immunological status. Furthermore, CCR, check-point, Inflammation promoting, Cytolytic activity, Parainflammation, T cell co-inhibition, T cell co-stimulation, and Type II IFN Response were also remarkably different in two groups (Fig. 10b). We investigated the variation in expression of immune checkpoints further. As shown in Fig. 10c, we revealed that most immune checkpoint molecule expression levels were noticeably higher in high-risk subgroups, which indicated that the immune checkpoint inhibitors have potential effectiveness for the patient’s therapy with high risk. ESTIMATE algorithm was conducted to calculate the density and location of immune cells in ccRCC cases to provide immune, stromal, and ESTIMATE scores. In comparison to low-risk group, the high-risk group had a higher immune score as well as ESTIMATE score (Fig. 10d–f).

### Prediction of drug sensitivity for FLPS

**Figure 11:**
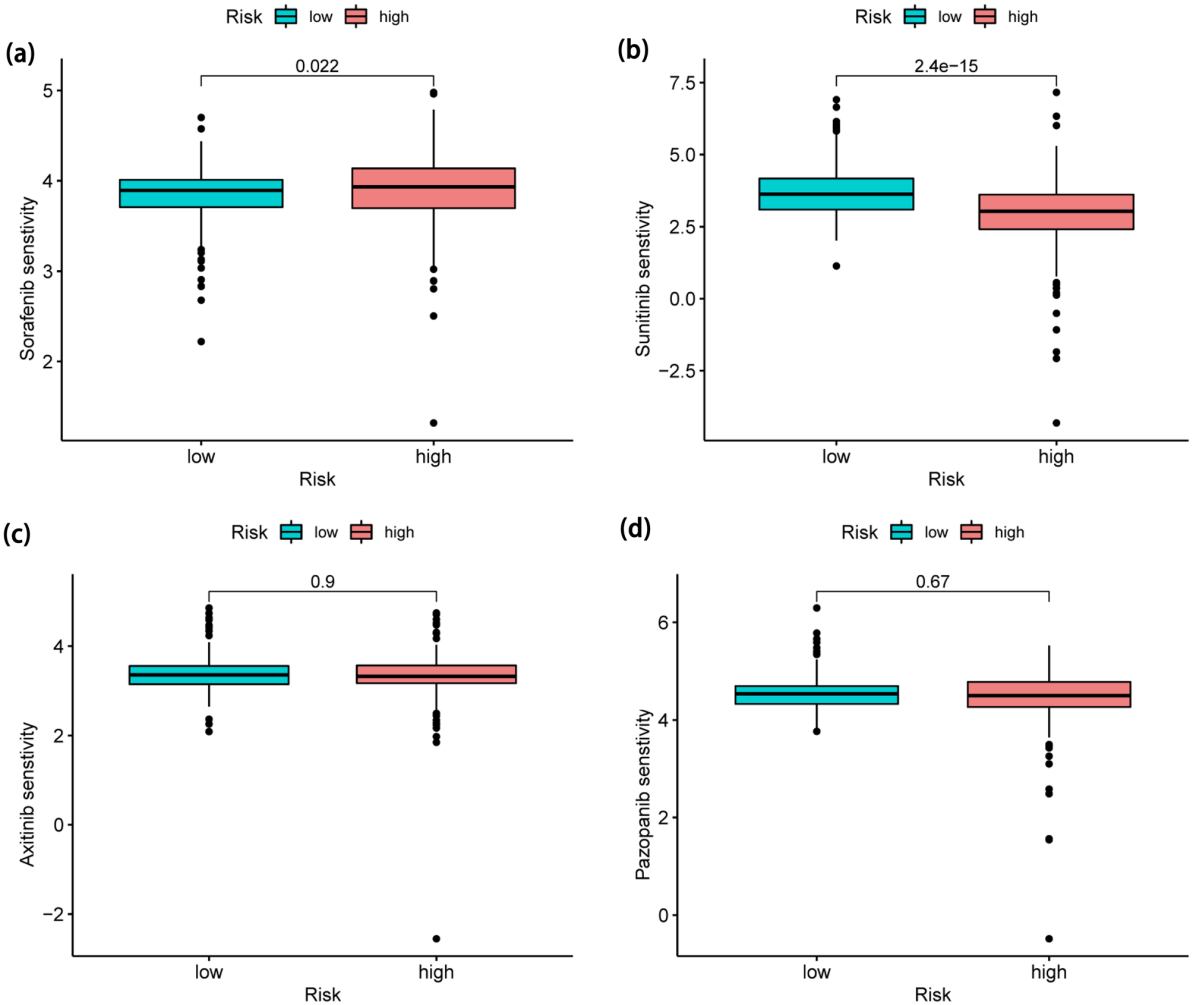
Differences in sensitivity of two groups to different drug responses. (**a**) Sorafenib (P = 0.022). (**b**) Sunitinib (P = 2.4e−15). (**c**) Axitinib (P = 0.9). (**d**) Pazopanib (P = 0.67).

Surprisingly, ccRCC with high risk were vulnerable to sorafenib (Fig. 11a), yet those in low-risk group were susceptible to sunitinib (Fig. 11b). However, neither of the two groups benefited from axitinib or pazopanib at all (Fig. 11c, d). These findings also suggested that individuals with various subtypes may show striking variations in their response to various chemotherapeutic drugs.

## Discussion

Only 2% of all malignancies are identified as Renal Cell Carcinoma (RCC), however, it has been increasing for the past 20 years. In the previous 15 years, the prognosis of metastatic RCC has greatly improved thanks to immunotherapies and antiangiogenic therapies^20^. Nevertheless, medication resistance that has developed over time challenges oncology therapy. Histologically, the primary subtype of RCC is Clear cell Renal Cell Carcinoma (ccRCC)^21^. We adopted ccRCC as our research subject for this reason. Previous research has demonstrated that individuals with the same TNM classification and risk factor are likely to experience variable clinical outcomes owing to molecular heterogeneity^6^. Thus, determining effective prognostic molecular characteristics is strongly warranted. A rare iron-dependent and nonapoptotic type of cell death are known as ferroptosis^7^. Currently, ferroptosis is regarded as an effective target for the elimination of malignant cells in cancer treatment^22^. The migration, invasion, as well as proliferation of many malignancies, are all associated with ferroptosis. It has been proven that lncRNA is strongly linked with the occurrence of cancer. Furthermore, lncRNA is the main factor in the ferroptosis of tumors, according to accumulating evidence^20^. Hence, FA-lncRNAs are probable prognostic factors.

Previous studies have examined the application of FLPS to predict prognosis in a variety of malignancies, such as pancreatic ductal adenocarcinoma^23^, gastric cancer^24^, and lung adenocarcinoma^25^. Hitherto only Feng Li et al.^6^ discovered three FA-lncRNAs, that is DUXAP8, LUCAT1, and LINC02609 were substantially associated with the OS of ccRCC independently. Hence, more investigation into the FA-lncRNA interaction of the ccRCC prognostic signature is required. It is well established that LncRNAs have a contribution to the immunological microenvironment of tumors in ccRCC^26–28^. Multiple pieces of evidence support the importance of ferroptosis in effectiveness of immunotherapy^29^. Therefore, we further clarified the function of FA-lncRNA in the immune microenvironment, based on the construction of prognostic signature.

In our work, difference and correlation analysis were utilized to screen a total of 433 FA-lncRNAs. Then, after filtering, we carried out univariate Cox regression, Lasso, and multivariate Cox regression analyses, sequentially. 8 FA-lncRNAs were determined and developed prognostic signatures. The KM curves demonstrated that the OS of ccRCC with high risk was shorter than that with low risk. The validation cohort confirmed the aforementioned results. The signature demonstrated high specificity and sensitivity, according to time-dependent ROC curves. Our investigation also showed that the FLPS may function as a standalone prognostic indicator. Nomogram could calculate risk scores and forecast the OS of ccRCC patients. Clinical characteristic correlation analysis added to the stability of our signature's verification.

Furthermore, the co-expression network and the Sankey diagram were constructed to investigate and visualize the correlation between 8 FALs and 20 FAGs. The expression and prognosis of two FALs (LINC00460 and LINC00551) and RRM2 target genes were also verified by The Gene Expression Profiling Interactive Analysis^30^ (GEPIA2, http://gepia2.cancer-pku.cn/#index) and The University of Alabama at Birmingham Cancer data analysis Portal^31^ (UALCAN, http://ualcan.path.uab.edu/index.html). Importantly, the interaction between FLPS and tumor immune microenvironment (TME) was examined in ccRCC. There was a marked difference in immune cells in two groups (P < 0.05). Moreover, ccRCC with high risk typically had greater immune, stromal, as well as ESTIMATE scores, which indicated that TME in high-risk group was superior to low-risk group. The latest evidence has shown that the combination of ferroptosis and immune checkpoint (ICI) have synergistically enhanced anti-tumor efficacy. Consequently, these lncRNAs associated with ferroptosis may be the target of combination therapy with immune checkpoint inhibitors. First reported by Wang et al.^32^, CD8 + T cells reduced the expression of SLC7A11 and SLC3A2 by releasing interferon γ (IFNγ), which increased iron-specific lipid peroxidation in carcinoma cells. In our research, the high-risk group had higher ICIs expression. For instance, a modest rise in CTLA-4 expression in high-risk group raises the likelihood that these patients might profit more from anti-CTLA-4 immunotherapy. Members with high risk were also more responsive to sorafenib, according to the results of our drug sensitivity study.

However, the present study still had a variety of limitations. Our FLPS has already undergone internal validation. Whereas, it is challenging to carry out external validation on account of proper datasets in Gene Expression Omnibus^33^ (GEO, https://www.ncbi.nlm.nih.gov/gds) and International Cancer Genome Consortium (ICGC, https://dcc.icgc.org/) for model’s validation could not be found. In addition, further experimental validation in vivo and in vitro on these eight lncRNAs is also warranted.

## Methods

### Data collection and processing

To recognize ccRCC-associated signatures, the RNA-seq transcriptome data which encompassed 539 ccRCC cases and 72 normal cases, and clinical information were acquired from TCGA^34^. Less than 30-day survival times for samples resulted in their disqualification, thus, 513 ccRCC patients in total were recruited for further survival analysis. 513 ccRCC patients in total were assigned to an experimental cohort and a validation cohort (257 patients vs 256 patients) at random using the "caret" software in a 1:1 ratio. Furthermore, 384 ferroptosis-associated genes (FAGs) were imported from FerrDb^35^ (http://www.zhounan.org/ferrdb/index.html), containing 167 drivers, 104 suppressors, and 113 markers (Supplementary Table S1). The correlation between ferroptosis-associated long noncoding RNAs (FALs) and FAGs was investigated using Pearson's correlation analysis and | Pearson's correlation coefficient | > 0.5 along with a P < 0.01 were considered statistically significant results. The "limma" R package also revealed ferroptosis-associated differentially expressed genes (FA-DEGs) and ferroptosis-associated differentially expressed lncRNAs (FA-DELs) (|Log2FoldChange| >2, FDR < 0.05).

### Development and verification of prognosis ferroptosis-associated lncRNAs signatures

Following filtering, 96 FA-DELs remained after applying Univariate Cox regression analysis to assess 433 FA-DELs, where statistical significance was determined to exist at P < 0.01. For overfitting avoidance, the least absolute shrinkage and selection operator (LASSO) analysis was implemented along with 10-fold cross validation. The package "glmnet" of R was applied to the analyses above. Additionally, multivariate regression analysis was used to assess potential lncRNA candidates and a risk evaluation model was constructed utilizing the equation as follows:

$$Risk score={\sum }_{n=1}^{\infty }\left(coefficient (FR-DEL{}_{n})\times exp (FR-DEL{}_{n})\right)$$

The median value of the risk score was used to develop subgroups for ccRCC patients. By employing the Kaplan-Meier (KM) survival curves, we were able to demonstrate the distinction between these two populations. The heatmaps and scatterplots were generated to visualize gene expression in different groups and predict prognosis. Moreover, the effectiveness of the predictions was evaluated utilizing ROC curves.

### Independent factors analysis and development of a predictive nomogram

To ascertain if the risk score and clinical traits (Age, Gender, Grade, and Tumor stage) were independent predictive variables of ccRCC, univariate and multivariate Cox regression analyses were then used. We then compared the precision of diverse variables in predicting the outcome using ROC. Furthermore, on account of risk values and additional clinicopathologic characteristics, a nomogram was developed for providing a trustworthy tool for the prediction of the fate of ccRCC patients.

The level of conformity between the anticipated and observed patient populations was then assessed using Calibration curves. When the calibration curve matches the standard curve, the nomogram's predictive performance will be enhanced. To develop the nomogram and calibration curve, we utilized the "regplot" and "rms" programs. Then, time-dependent ROC curve investigations were carried out employing the "timeROC" program.

### Establishment of a co-expression network and confirmation

The correlation between 8 FALs and FAGs was investigated using Pearson's correlation analysis by conducting the “limma” package and | Pearson's correlation coefficient | > 0.5 along with a P < 0.01 were considered as there was a good correlation between 8 FALs and FAGs. For the purpose of displaying the network between 8 FALs and associated FAGs, Cytoscape version 3.8.2 was performed. Furthermore, major FALs and FAGs were further verified by other databases. The Gene Expression Profiling Interactive Analysis^30^ (GEPIA2, http://gepia2.cancer-pku.cn/#index), which comprised RNA-seq and corresponding clinical information provided by TCGA database and GTEx. The University of Alabama at Birmingham Cancer data analysis Portal^31^ (UALCAN, http://ualcan.path.uab.edu/index.html) was conducted to investigate FAGs.

### Function enrichment analysis

Gene set enrichment analysis (GSEA) was used to recognize possible functional pathways related to ferroptosis-associated lncRNA prognostic signature (FLPS)^36^. In addition, identifying the substantial enrichment pathways in both two groups is of great significance. Thus, according to optimal cutoff values, we also divided TCGA samples into low- and high-risk groups. GSEA v. 4.1.0, C2.cp.kegg.v7.5.symbols.gmt were employed, with |NES|≥1, FDR < 0.25 as well as P < 0.05 being recognized as statistically significant.

### Immunity analysis

To assess the cellular immunological response of low- and high-risk groups, we retrieved Infiltrate immune cell data from TIMER2.0 (http://timer.cistrome.org/)^37^, which incorporated 6 new algorithms, including CIBERSORT, MCP-counter, quanTIseq, EPIC, xCell, as well as TIMER. In addition, the activity of 13 immune-related pathways was determined by single-sample Gene Set Enrichment Analysis (ssGSEA) algorithms^38^. Immune, stromal, as well as ESTIMATE scores, were determined via ESTIMATE algorithm. These scores indicated the proportions of these components in the tumor microenvironment (TME)^39^, along with their immune and stromal component ratios^40^. Additionally, the possible immunological checkpoint was obtained from prior studies and applied to assess the distinction between these two groups^41^.

### Drug sensitivity prediction

The first-line therapy medications sorafenib, sunitinib, pazopanib, and axitinib were assessed on patients in two groups employing "pRRophetic" package. The Wilcoxon signed-rank test was used to examine the disparity between the two groups.

### Analytical statistics

R v. 4.1.0 was applied to all statistical and computational investigations. To examine relationships between the clinicopathologic parameters and FLPS, the Wilcoxon rank-sum test was used. All differences were significant when P < 0.05.

## Supplementary Information

Supplementary Tables.

## Data Availability

The datasets TCGA-KIRC and 384 ferroptosis-associated genes (FAGs) can be extracted from the TCGA database (https://tcga-data.nci.nih.gov/tcga/)^24^, and FerrDb^35^ database (http://www.zhounan.org/ferrdb/index.html).
