# Supplementary material for: Ferroptosis-associated lncRNA prognostic signature predicts prognosis and immune response in clear cell renal cell carcinoma
Source: Sci Rep. 2023 Feb 6;13:2114. doi: 10.1038/s41598-023-29305-5 (PMC9902540; doi:10.1038/s41598-023-29305-5)
Supplement: Supplementary file 1 — Supplementary Tables. [file 41598_2023_29305_MOESM1_ESM.pdf]

Ferroptosis-Associated lncRNA Prognostic Signature Predicts Prognosis and Immune Response in Clear Cell Renal Cell Carcinoma

Jiayi Lai[1], Shiqi Miao[1], Longke Ran[1]*

[1]Department of Bioinformatics, The Basic Medical School of Chongqing Medical University, Chongqing, 400016, China.

*corresponding. ranlongke@cqmu.edu.cn

Supplementary Material

1 Supplementary Tables

1.1 Supplementary Table S1. A list of ferroptosis-related genes

RPL8
IREB2
ATP5MC3
CS
EMC2
ACSF2
NOX1
CYBB
NOX3
NOX4
NOX5
DUOX1
DUOX2
G6PD
PGD
VDAC2
PIK3CA

FLT3
SCP2
TP53
ACSL4
LPCAT3
NRAS
KRAS
HRAS
TF
TFRC
TFR2
SLC38A1
SLC1A5
GLS2
GOT1
CARS1
ALOX5
KEAP1
HMOX1
ATG5
ATG7
NCOA4
ALOX12
ALOX12B
ALOX15
ALOX15B
ALOXE3
PHKG2
ACO1
G6PDX
ULK1
ATG3
ATG4D
BECN1
MAP1LC3A
GABARAPL2

GABARAPL1
ATG16L1
WIPI1
WIPI2
SNX4
ATG13
ULK2
SAT1
EGFR
MAPK3
MAPK1
BID
ZEB1
DPP4
CDKN2A
PEBP1
SOCS1
CDO1
MYB
MAPK8
MAPK9
CHAC1
MAPK14
LINC00472
PRKAA2
PRKAA1
ELAVL1
BAP1
ABCC1
MIR6852
ACVR1B
TGFBR1
EPAS1
HILPDA
HIF1A
IFNG
ANO6

LPIN1
HMGB1
TNFAIP3
TLR4
ATF3
ATM
YY1AP1
EGLN2
MIOX
TAZ
MTDH
IDH1
SIRT1
FBXW7
PANX1
DNAJB6
BACH1
LONP1
CD82
IL1B
CTSB
POR
CYB5R1
ELOVL5
FADS1
FBW7
PTEN
NR1D1
NR1D2
TBK1
IL6
USP7
miR-182-5p
miR-378a-3p
ATF4
AQP3

AQP5
AQP8
LINC00618
MT1DP
PEX10
AGPAT3
PEX12
CHP1
GPAT4
BRPF1
OSBPL9
INTS2
MMD
CYP4F8
MLLT1
TTPA
GRIA3
EPT1
POM121L12
LIG3
AEBP2
AGPS
CDCA3
PEX2
PEX6
TIMM9
DCAF7
LCE2C
FAR1
PHF21A
SMAD7
LYRM1
AMN
PEX3
MTCH1
ACADSB
PVT1

hsa_circ_0008367
SLC39A14
MAP3K11
GSK3B
SLC7A11
GPX4
AKR1C1
AKR1C2
AKR1C3
RB1
HSPB1
HSF1
GCLC
NFE2L2
SQSTM1
NQO1
HMOX1
FTH1
MUC1
SLC3A2
MT1G
SLC40A1
CISD1
FANCD2
FTMT
HSPA5
ATF4
TP53
HELLS
SCD
FADS2
SRC
STAT3
PML
MTOR
NFS1

TP63
CDKN1A
MIR137
ENPP2
VDAC2
FH
CISD2
MIR9-1
MIR9-2
MIR9-3
CBS
ISCU
ACSL3
OTUB1
CD44
LINC00336
BRD4
PRDX6
MIR17
SESN2
NF2
ARNTL
HIF1A
JUN
CA9
TMBIM4
PLIN2
MIR212
Fer1HCH
AIFM2
LAMP2
ZFP36
PROM2
CHMP5
CHMP6
CAV1
GCH1

SIRT3
DAZAP1
PIR
FTL
HCAR1
SLC16A1
RRM2
NR4A1
PIK3CA
RPTOR
SREBF1
SREBF2
FZD7
P4HB
NT5DC2
BCAT2
PLA2G6
MIR424
PARK7
FXN
SUV39H1
ATF2
ACOT1
ALDH3A2
STK11
FNDC5
CircIL4R
CDH1
MIR214
NEDD4L
TF
BRD2
BRD3
BRDT
DECR1
PTGS2

DUSP1
NOS2
NCF2
MT3
UBC
ALB
TXNRD1
SRXN1
GPX2
BNIP3
OXSR1
SELENOS
ANGPTL7
CHAC1
SLC7A11
DDIT4
LOC284561
ASNS
TSC22D3
DDIT3
JDP2
SESN2
SLC1A4
PCK2
TXNIP
VLDLR
GPT2
PSAT1
LURAP1L
SLC7A5
HERPUD1
XBP1
ATF3
SLC3A2
CBS
ATF4
ZNF419

KLHL24
TRIB3
ZFP69B
ATP6V1G2
VEGFA
GDF15
TUBE1
ARRDC3
CEBPG
SNORA16A
RGS4
BLOC1S5-TXNDC5
LOC390705
EIF2S1
KIM-1
IL6
CXCL2
RELA
HSD17B11
AGPAT3
SETD1B
HMOX1
TF
FTL
RPL8
ATP5MC3
TFRC
MAFG
IL33
FTH1
SLC40A1
GPX4
HAMP
HSPB1
NFE2L2

STEAP3
DRD5
DRD4
MAP3K5
MAPK14
SLC2A1
SLC2A3
SLC2A6
SLC2A8
SLC2A12
GLUT13
SLC2A14
EIF2AK4
ALOX5
ALOX12
ALOX15
ACSF2
IREB2
HMGB1
ELAVL1
TFAP2C
SP1
HBA1
NNMT
PLIN4
HIC1
STMN1
RRM2
CAPG
HNF4A
NGB
YWHAE
GABPB1
AURKA
MIR4715
RIPK1
PRDX1

MIR30B
MMP13
LRRFIP1

1.2 **Supplementary Table S2.** Univariate Cox regression analysis identified 206 significant FR-DELs

id	HR	HR.95L	HR.95H	pvalue
AL161452.1	2.974066	1.37062	6.453334	0.005823
AC008105.2	1.47242	1.097032	1.976259	0.009974
AC244197.2	1.632917	1.189613	2.241416	0.00241
AP005233.2	1.242314	1.092345	1.412872	0.000948
Z99289.1	2.76151	1.514479	5.035356	0.000919
AP000553.2	1.719081	1.169216	2.527539	0.005871
AL513218.1	2.311876	1.525637	3.503305	7.76E-05
AC005899.7	2.882625	1.60798	5.167681	0.000378
AC073218.1	1.295682	1.103897	1.520786	0.001528
AL596223.2	2.162316	1.254454	3.727208	0.005503
AC093788.1	2.479539	1.658085	3.707961	9.74E-06
MIAT	1.505182	1.224877	1.849633	0.000101
LINC01705	1.461374	1.182328	1.806279	0.00045
AC010973.2	1.999346	1.380503	2.895599	0.000246
AC078906.1	2.245132	1.373913	3.668806	0.001248
AC107081.1	1.888135	1.286411	2.771319	0.001169
YEATS2-AS1	3.201574	1.839295	5.57283	3.87E-05
AL139123.1	3.205642	1.762465	5.83055	0.000135
N4BP2L2-IT2	1.968715	1.282592	3.021881	0.001946
U47924.3	2.186345	1.407491	3.396187	0.000499
NARF-IT1	2.491381	1.434598	4.326634	0.001189
AC005785.1	2.813972	1.806207	4.384016	4.79E-06
PTOV1-AS2	1.405998	1.116434	1.770666	0.003779
AC008735.2	1.331724	1.083331	1.63707	0.00653
AL645939.4	1.711409	1.17095	2.50132	0.00552
KIF1C-AS1	2.715942	1.681635	4.386408	4.41E-05
AC008875.1	2.631528	1.641386	4.218959	5.88E-05
AC012615.6	1.75638	1.196044	2.579229	0.004064
AL135999.1	1.706983	1.191813	2.444838	0.003531

AC027796.4	1.72945	1.310946	2.281557	0.000106
AC092119.2	1.944666	1.306797	2.893888	0.001041
LINC00460	1.642665	1.36274	1.980089	1.92E-07
AC005387.1	2.299164	1.463176	3.612795	0.000305
MYG1-AS1	1.80892	1.37767	2.375164	1.99E-05
AC124854.1	0.703454	0.594081	0.832963	4.51E-05
AC004034.1	2.944876	1.614326	5.372084	0.000429
AC008870.2	2.776636	1.743599	4.42172	1.69E-05
AC084876.1	2.401392	1.689087	3.414083	1.06E-06
AC011510.1	2.068788	1.286406	3.327008	0.002709
ASMTL-AS1	1.312705	1.099061	1.567878	0.002681
AC005840.2	1.999934	1.341171	2.982273	0.000674
AC020907.4	1.771878	1.321579	2.375607	0.000131
AL122125.1	1.922817	1.23311	2.998291	0.003921
AL731567.1	1.741086	1.280901	2.3666	0.000399
PVT1	1.632689	1.188641	2.242624	0.00247
LINC01871	1.549969	1.186023	2.025596	0.00133
AC025265.1	1.470627	1.152644	1.876333	0.001917
LINC00894	2.193152	1.417276	3.393775	0.000423
AL157392.4	2.099479	1.340465	3.28827	0.001195
LINC01355	2.073305	1.481771	2.900985	2.10E-05
AL021707.6	1.421102	1.114105	1.812693	0.004653
LINC01943	1.901618	1.259442	2.871231	0.002234
IGFL2-AS1	1.358858	1.130346	1.633567	0.001098
SLC16A1-AS1	1.6461	1.134319	2.388786	0.008708
AC006435.2	1.539163	1.11258	2.129306	0.009209
LINC00551	0.076585	0.01089	0.53859	0.00983
AC005387.2	1.985843	1.28332	3.072945	0.002071
CD44-AS1	3.96614	2.007374	7.83624	7.32E-05
NFE4	1.521668	1.235926	1.873473	7.62E-05
AP001029.1	1.465924	1.108581	1.938455	0.007295
AC027601.1	3.886211	1.885333	8.010592	0.000235
AC083967.1	1.511646	1.159463	1.970804	0.002264
AC026356.2	1.685565	1.139557	2.493188	0.008948
AC132872.3	1.460141	1.186035	1.797596	0.000359
SLBP-DT	2.225545	1.562558	3.169833	9.28E-06
LINC00893	1.667884	1.170313	2.377001	0.004655

AC092809.4	2.471412	1.454353	4.199719	0.000824
AC025171.4	1.567208	1.217218	2.017833	0.000493
AL158151.4	2.718176	1.73488	4.258785	1.27E-05
LINC00342	1.446305	1.142227	1.831333	0.002183
AC040162.3	2.721964	1.520085	4.874127	0.000755
AL353804.2	2.16918	1.214928	3.872937	0.008839
AC110015.1	2.464795	1.383864	4.390037	0.002191
MIR155HG	1.366897	1.117236	1.672347	0.002387
AL391056.1	1.607691	1.195618	2.161787	0.001675
AC011462.4	1.492713	1.169497	1.905256	0.001293
UBE2Q1-AS1	2.636517	1.513232	4.593625	0.000621
AL096865.1	2.158528	1.418372	3.284924	0.000329
AC005104.1	1.630956	1.189693	2.235885	0.002373
TCL6	0.343111	0.154298	0.762974	0.008705
AC005306.1	2.525816	1.439517	4.431866	0.001238
KLHDC7B-DT	1.50855	1.147729	1.982807	0.0032
AC012645.4	1.882288	1.172956	3.02058	0.008766
AL158834.2	4.21369	2.119213	8.378196	4.10E-05
AC018648.1	6.0751	3.157622	11.68817	6.52E-08
AC245884.8	1.394151	1.083544	1.793795	0.009769
LINC02154	1.758993	1.338109	2.312261	5.18E-05
AC073487.1	2.365407	1.508848	3.708227	0.000175
AC006272.1	3.096167	1.523361	6.292827	0.001789
LINC00926	2.238774	1.427895	3.510139	0.000444
AC044781.1	2.841004	1.333687	6.051874	0.006804
AC105105.3	1.823502	1.17008	2.841822	0.007959
AL023653.1	2.424227	1.365172	4.304863	0.002508
LINC02446	1.762699	1.305482	2.380046	0.000216
AC084117.1	1.301853	1.067342	1.587891	0.009239
TMEM92-AS1	1.933177	1.374877	2.718187	0.00015

1.3 **Supplementary Table S4.** Correlation analysis between 8 FALs and 25 FAGs.

ferrGene	lncRNA	cor	pvalue	regulation
CDKN2A	LINC00460	0.52895831	3.45E-40	postive

CDCA3	LINC00460	0.564229432	1.24E-46	postive
RRM2	LINC00460	0.572292088	3.23E-48	postive
STMN1	LINC00460	0.520268565	1.03E-38	postive
AURKA	LINC00460	0.542306356	1.54E-42	postive
PRKAA2	AC124854.1	0.50757467	1.23E-36	postive
PHKG2	AC084876.1	0.510014502	4.98E-37	postive
TAZ	AC084876.1	0.629575722	7.49E-61	postive
PVT1	AC084876.1	0.563040824	2.11E-46	postive
TUBE1	AC084876.1	0.54044266	3.34E-42	postive
IDH1	IGFL2-AS1	0.518959048	1.70E-38	postive
TFAP2C	LINC00551	0.532785702	7.50E-41	postive
JDP2	AC083967.1	0.518155329	2.31E-38	postive
ALOX12	AC073487.1	0.598650425	1.03E-53	postive
ATM	AC073487.1	0.663168673	1.40E-69	postive
FBXW7	AC073487.1	0.52151505	6.36E-39	postive
FAR1	AC073487.1	0.504926058	3.26E-36	postive
HELLS	AC073487.1	0.534353765	3.99E-41	postive
ZNF419	AC073487.1	0.506121796	2.10E-36	postive
TUBE1	AC073487.1	0.597180622	2.15E-53	postive
GABPB1	AC073487.1	0.582983593	2.19E-50	postive
CDKN2A	LINC02446	0.507301079	1.36E-36	postive
RRM2	LINC02446	0.547962959	1.45E-43	postive

STMN1	LINC02446	0.599655647	6.19E-54	postive
AURKA	LINC02446	0.505422101	2.72E-36	postive

1.4 **Supplementary Table S3.** GESA enrichment analysis of low-risk group

GS<br>follow link to MSigDB	NES	NOM p-val	FDR q-val
KEGG_PROXIMAL_TUBULE_BICARBONATE_RECLAMATION	-2.30262	0	0.031591
KEGG_PROPANOATE_METABOLISM	-2.24592	0	0.02516
KEGG_VASOPRESSIN_REGULATED_WATER_REABSORPTION	-2.22411	0	0.020912
KEGG_VALINE_LEUCINE_AND_ISOLEUCINE_DEGRADATION	-2.21725	0	0.017044
KEGG_TRYPTOPHAN_METABOLISM	-2.2079	0	0.01542
KEGG_PEROXISOME	-2.1817	0	0.01634
KEGG_TIGHT_JUNCTION	-2.18089	0	0.014006
KEGG_PYRUVATE_METABOLISM	-2.17093	0	0.013464
KEGG_GLYCOLYSIS_GLUCONEOGENESIS	-2.15664	0.001957	0.013841
KEGG_FATTY_ACID_METABOLISM	-2.13667	0	0.015431
KEGG_BUTANOATE_METABOLISM	-2.12323	0	0.016271
KEGG_RENIN_ANGIOTENSIN_SYSTEM	-2.1207	0	0.015545
KEGG_CITRATE_CYCLE_TCA_CYCLE	-2.0693	0	0.024474
KEGG_GLYCINE_SERINE_AND_THREONINE_METABOLISM	-2.02991	0.003891	0.030817
KEGG_BETA_ALANINE_METABOLISM	-1.98816	0.011696	0.040035
KEGG_ADIPOCYTOKINE_SIGNALING_PATHWAY	-1.97945	0.004065	0.040569
KEGG_TERPENOID_BACKBONE_BIOSYNTHESIS	-1.96563	0.005792	0.042471
KEGG_SPHINGOLIPID_METABOLISM	-	0.004082	0.045782

	1.95021		
KEGG_ADHERENS_JUNCTION	-1.94398	0.020121	0.045267
KEGG_PROSTATE_CANCER	-1.93329	0.014141	0.045865
KEGG_PPAR_SIGNALING_PATHWAY	-1.91692	0.009615	0.048701
KEGG_INSULIN_SIGNALING_PATHWAY	-1.91522	0.00994	0.046881
KEGG_ENDOMETRIAL_CANCER	-1.89299	0.015968	0.051521
KEGG_BIOSYNTHESIS_OF_UNSATURATED_FATTY_ACIDS	-1.88942	0.013807	0.050919
KEGG_RENAL_CELL_CARCINOMA	-1.86274	0.023857	0.057605
KEGG_ENDOCYTOSIS	-1.85947	0.015779	0.056331
KEGG_ERBB_SIGNALING_PATHWAY	-1.85545	0.018145	0.055501
KEGG_CYSTEINE_AND_METHIONINE_METABOLISM	-1.85053	0.009363	0.055201
KEGG_NEUROTROPHIN_SIGNALING_PATHWAY	-1.84952	0.025845	0.053575
KEGG_LYSINE_DEGRADATION	-1.83391	0.019646	0.057789
KEGG_ONE_CARBON_POOL_BY_FOLATE	-1.8148	0.015534	0.063084
KEGG_TYPE_II_DIABETES_MELLITUS	-1.81283	0.001965	0.061854
KEGG_ARGININE_AND_PROLINE_METABOLISM	-1.80381	0.017143	0.063466
KEGG_HISTIDINE_METABOLISM	-1.79733	0.021318	0.064003
KEGG_GLYCOSYLPHOSPHATIDYLINOSITOL_GPI_ANCHOR_BIOSYNTHESIS	-1.79056	0.014028	0.064847
KEGG_MELANOMA	-1.77995	0.00432	0.067613
KEGG_ALDOSTERONE_REGULATED_SODIUM_REABSORPTION	-1.76672	0.02008	0.071079
KEGG_TGF_BETA_SIGNALING_PATHWAY	-1.7504	0.042596	0.076175
KEGG_THYROID_CANCER	-1.73795	0.029126	0.079386
KEGG_LONG_TERM_POTENTIATION	-1.73666	0.024145	0.077838

KEGG_GLYCEROLIPID_METABOLISM	-1.70727	0.015779	0.0888
KEGG_LONG_TERM_DEPRESSION	-1.70623	0.025794	0.087264
KEGG_SELENOAMINO_ACID_METABOLISM	-1.66222	0.036437	0.104484
KEGG_ASCORBATE_AND_ALDARATE_METABOLISM	-1.66143	0.033708	0.1026
KEGG_EPITHELIAL_CELL_SIGNALING_IN_HELICOBACTER_PYLORI_INFECTION	-1.6531	0.047151	0.104157
KEGG_PANTOTHENATE_AND_COA_BIOSYNTHESIS	-1.64588	0.04771	0.098978
KEGG_PHENYLALANINE_METABOLISM	-1.59327	0.02994	0.110038
KEGG_NITROGEN_METABOLISM	-1.55726	0.040486	0.117068
KEGG_DRUG_METABOLISM_CYTOCHROME_P450	-1.55388	0.034091	0.116979
KEGG_ETHER_LIPID_METABOLISM	-1.50884	0.035156	0.122849
